# Effect of Sonic Application of Self-Adhesive Resin Cements on Push-Out Bond Strength of Glass Fiber Posts to Root Dentin

**DOI:** 10.3390/ma12121930

**Published:** 2019-06-14

**Authors:** Darlon Martins Lima, Thátyla Silva Linhares, Suellen Nogueira Linares Lima, Edilausson Moreno Carvalho, Alessandro Dourado Loguercio, José Bauer, Ceci Nunes Carvalho

**Affiliations:** 1 Department of Dentistry I, School of Dentistry, University Federal of Maranhão, São Luis, Maranhão 65080-805, Brazil; darlonmartins@yahoo.com.br (D.M.L.); linhares.thatyla@yahoo.com.br (T.S.L.); 2 Department of Restorative Dentistry, School of Dentistry, Ceuma University (Uniceuma), São Luis, Maranhão 65065-470, Brazil; suellenlinareslima@gmail.com (S.N.L.L.); ceci.carvalho@ceuma.br (C.N.C.); 3 Discipline of Dental Materials, School of Dentistry, University Federal of Maranhão, São Luis, Maranhão 65080-805, Brazil; edilausson@gmail.com; 4 Department of Restorative Dentistry, School of Dentistry, State University of Ponta Grossa (UEPG), Ponta Grossa, Paraná 84.010-170, Brazil; aloguercio@hotmail.com

**Keywords:** self-adhesive resin cements, fiber post, dentin, push-out bond strength, sonic application

## Abstract

The aim of this study was to evaluate the influence of a sonic application of self-adhesive resin cements on the bond strength of glass fiber posts to root dentin. Eighty bovine incisors were randomly divided into eight groups (*n* = 10). Four self-adhesive resin cements were used—RelyX U200 (3M/ESPE), Bifix SE (Voco), seT PP (SDI), and Panavia SA (Kuraray). The cements were inserted into the root canal in two different modes—Centrix syringe (control) or with a sonic device (Sonic Smart). The roots were sectioned and taken to a universal test machine (Instron 3342) to perform the push-out test. The fracture pattern was evaluated by stereomicroscope and scanning electron microscope. The bond strength data were analyzed by two-way ANOVA and Tukey tests (α = 0.05). The interaction between the main factors was significant (*p* = 0.002). The sonic application increased the bond strength in comparison with the conventional application for the RelyX U200 (*p* < 0.001) and Bifix SE (*p* < 0.017) cements. However, for the seT PP and Panavia SA cements, the bond strength values did not differ significantly (*p* > 0.05). The fracture pattern showed adhesive at the interface between the luting cement and the dentin. Using a sonic device in the application of self-adhesive resin cement helpedpromote an increase in the bond strength for RelyX U200 and Bifix SE.

## 1. Introduction

Some resin cements require acid etching of the dental hard tissues, and the use of an adhesive system requires numerous operating steps (e.g., acid etching and application of the primer and adhesive) with a longer working time, in addition to being more technique sensitive because of these various steps [1,2,3]. With the purpose of simplifying the cementation technique, self-adhesive resin cements were introduced, as they require a lower number of clinical steps [4,5]. Another advantage of using these materials is the increased control of the humidity of the substrate [6] because there is no need to remove the smear layer [7]. The bond of these cements is performed by acidic monomers that have a high affinity for the calcium of hydroxyapatite [5,8].

Despite the development of self-adhesive cements, the cementation of glass fiber posts (GFPs) within the root canal continues to be challenging. Usually, the material is inserted with the use of a microbrush, endodontic files, exploratory probes, or the GFP itself, which may lead to the appearance of failures in the interfacial layer [9]. To overcome this problem, the use of the Centrix syringe is one of the techniques most frequently used. This device is capable of diminishing the number of bubbles present at the interface of the material, which may influence the self-adhesive resin cement’s bond with the GFPs [10]. Nevertheless, during cementation, the resin cement must be applied under pressure to guarantee intimate contact between the cement and the dentin. When this pressure is inadequate, there is deficient adaptation and a low bond strength [8].

However, new techniques have been developed as a way to minimize the failures in this operating step, such as the use of devices for sonic application [11]. Recently, some authors have shown that the active application of universal adhesives in self-etch mode to enamel appears as a viable alternative to selective enamel etching [12]. Recently, an in vitro study showed that application of the adhesive systems with a sonic device oscillating at 170 Hz improved the bond strength of adhesive systems to coronal [11] and root dentin [13].When adhesive materials are applied under sonic vibration, the fluids penetrate into areas where the applicator bristles do not reach and vibratory waves are created in the adhesive, which generate a greater diffusion of the material into the dentin substrate [13,14]. Furthermore, bubbles present in the adhesive material are pushed against the dentin walls and burst [13]. In addition, cements that have a high viscosity can increase their fluidity through the energy generated by sonic vibration [15]. However, the effectiveness of this technique, which may improve the interaction of self-adhesive resin cements with root dentin, for the cementation of intraradicular posts has not yet been investigated.

Therefore, the aim of the present study was to evaluate the influence, in vitro, of the sonic insertion of self-adhesive resin cements on the GFP bond strength. The null hypothesis tested was that the use of sonic application would not interfere with the bond strength of self-adhesive resin cements.

## 2. Materials and Methods

### 2.1. Preparation of Specimens

Eighty bovine incisors with straight roots, closed apices, and lengthsof 40 mm (+1.0) were used. The teeth were radiographed and curved or atresic canals were excluded. The crowns were sectioned below the cement–enamel junction by using a diamond-coated disk (KG Sorensen, Cotia, Brazil) at low speed under cooling to standardize the root length to 15 mm (±0.1). The coronal diameters of the root canals were measured in the mesio–distal and vestibular–lingual directions, with a digital caliper (Mitutoyo, Suzano, Brazil). Root canals with a coronal diameter equal to 2 mm (±0.1) were selected. The specimens were randomly allocated to eight experimental groups (*n* = 10), according to the type of cement and cement insertion technique used.

### 2.2. Post Cementation

A single operator, specialist in endodontics and who experienced all of the techniques used in this study, performed the root canal preparation, obturation, and post cementation procedures. Cleaning and shaping were performed using a crown-down canal root preparation technique using K-type files #80 (DentsplyMaillefer, Ballaigues, Switzerland). Working length was set at 1 mm from the apical foramen. Apical enlargement was performed with an instrument size of up to No. 70. Then the specimens were washed with 6% sodium hypochlorite solution and 17% EDTA (BiodinâmicaQuímica e Farmacêuitca Ltda., Ibiporã, PR, Brazil) for 5 min.

The root canals were obturated by lateral condensation of the gutta percha cones (Dentsply Sirona, York, PA, USA) and the calcium hydroxide cement (Sealer 26; Dentsply Sirona, York, PA, USA) and were prepared with a specific burr for the glass fiber post (GFP) system used (White Post DC #1, FGM, Joinville, Brazil), which was inserted up to the length of 9 mm in a single movement. 

Dentin debris and gutta percha were removed with distilled water, followed by drying with absorbent paper tips (Dentsply Maillefer, Ballaigues, Switzerland). For the root canal standardization, the drill corresponding to the glassfiber post No. 1 was used. After the removal of the gutta percha, the glassfiber posts were placed in the post space to assess the adaptation of the posts. Before the adhesion process, the GFPs were cleaned with 70% alcohol. After this, a layer of silane was applied (Prosil, FGM, Joinville, Brazil) on the post surfaces, with the help of an applicator brush (FGM, Joinville, Brazil). The cements were mixed in accordance with the manufacturers’ recommendations (Table 1).

**Figure 1:**
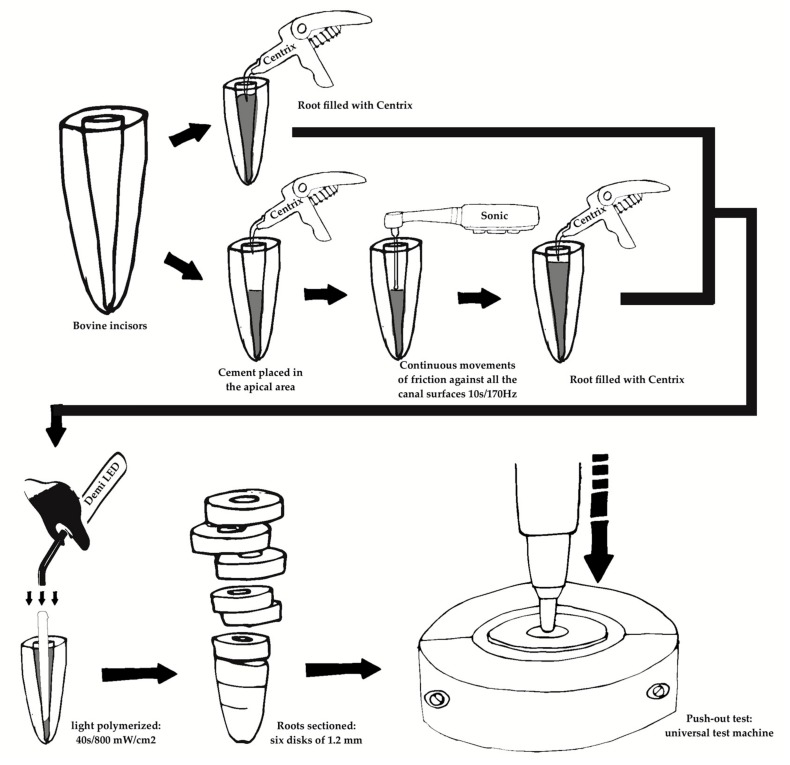
Schematic illustrating the root filling, cementation procedures of the post and push-out test.

In the groups in which the sonic device (Smart, FGM, Joinville, Brazil) was used, the first portion of cement was inserted into Centrix syringe (DFL, Rio de Janeiro, Brazil) and the cement was placed in the apical area (Figure 1). After this, a microbrush (Cavibrush regular, FGM, Joinville, Brazil) was attached on Sonic device andpositioned on the root dentin walls in continuous movements of friction against all the canal surfaces (vestibular, mesial, distal, and lingual) for a period of 10 s and a frequency of 170 Hz [11] (Figure 1). A finalportion of cement was inserted with a Centrix syringe, and the posts (DC #1, FGM, Joinville, Brazil) were inserted immediately, and light polymerized(Demi LED; Kerr Corporation, Orange, CA, USA) for 40 s with an intensity of 800 mW/cm^2^ (Demetron L.E.D. Radiometer; Kerr, Orange, CA, USA) with the light source positioned in the coronal portion of the root (Figure 1).

### 2.3. Push-Out Bond Strength Test

The roots were sectioned perpendicular to their long axis into six disks of 1.2 mm (±0.1) (i.e., six slices per root), with a double-faced diamond disk coupled to a cutting machine (Isomet 5000, Buehler, Lake Bluff, Illinois, USA) under constant cooling (Figure 1). 

The coronal and apical sides of each slice were photographed using a stereomicroscope (Kozo Optical and Electronic Instruments, Nanjing, China) at 30× magnification. The coronal and apical diameters of the root canal of each slice were measured with the aid of software (Image J, version 1.46, National Institutes of Health, Bethesda, MD, USA), and the thickness was measured with a digital caliper. 

The push-out test was performed in a universal test machine (Instron 3342, Canton, MA, USA) at a speed of 1.0 mm/min until the post was extruded (Figure 1). For each slice, the loading rod diameter was selected so that it could be 0.2 mm smaller than the apical diameter, in order to prevent it from touching the dentin walls during the test. The bond strength value, expressed in MPa, was calculated by the formula F/A, in which ‘F’ is the maximum force before the rupture of the interface, recorded by the universal testing machine in Newtons (N), and ‘A’ is the bonded interface area in millimeters.

The dentin/post interface area was calculated using the truncated cone area formula, $\left(R+r\right){\left[h^{2}+{\left(R-r\right)}^{2}\right]}^{0.5}$, in which π is a constant equal to 3.14, *h* is the slice height, and *R* is the greatest and *r* is the smallest post radius, and obtained on the coronal and apical diameters of each slice, respectively.

### 2.4. Stereomicroscope Pattern Evaluation (Fracture Pattern)

After the mechanical test, the specimens were evaluated under a stereomicroscope at 40× magnification (Kozo Optical and Electronic Instruments, Nanjing, China). The failures were classified into five types [16]: (1) adhesive between the self-adhesive resin cement and post, (2) adhesive between the resin cement and root dentin, (3) mixed, (4) cohesive in dentin, and (5) cohesive in post.

### 2.5. Scanning Electron Microscopy (SEM)

Test specimens representative of each type of fracture, resulting from the push-out test, were selected for SEM analysis For SEM examinations, the specimens were dried in a dessicator at room temperature (25° C) for 48 h. The opposing sides of the slice were bonded with double-sided carbon taped and then observed with an acceleration voltage of 15 kV in vacuum using SEM (TM 3030, Hitachi, Tokyo, Japan).The surfaces were observed in different locations at ×100 and ×250 magnifications in backscattered electrons mode [17].

### 2.6. Statistical Analysis

The experimental unit in this study was the root. The push-out bond strength test result of all slices from the same root was averaged for statistical purposes. The statistical analysis was performed by using Sigma Plot 13.0 (Systat Software Inc., San Jose, CA, USA). After observing the normality of the data distribution (Shapiro–Wilk test), the statistical analysis was performed by two-way analysis of variance (application mode vs. resin cements) and Tukey tests (α = 0.05). 

## 3. Results

### 3.1. Push-Out Bond Strength Test

**Figure 2:**
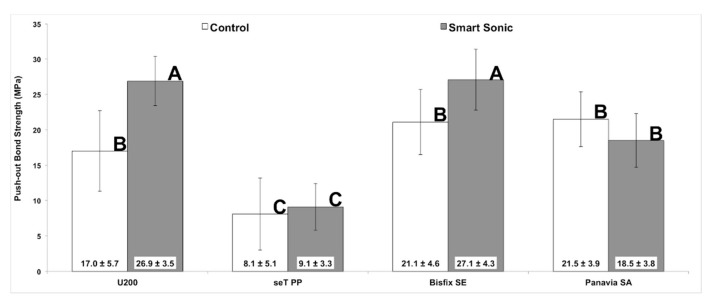
Mean and standard deviation of push-out bond strength values (MPa) in all the conditions evaluated. Different letters indicate statistical difference between the groups (*p* < 0.05).

The Figure 2 shows the means and standard deviations (MPa) obtained with the self-adhesive cements in all the experimental conditions evaluated. The analysis of variance demonstrated significant interaction between the main factors (*p* = 0.002). In the groups where RelyX U200 and Bifix SE cements were used with sonic insertion, there were significantly higher bond strength values than those obtained with a conventional application (*p* < 0.001). However, Panavia SA and seT PP cements presented similar bond strength values, irrespective of the application mode used (*p* > 0.5).

### 3.2. Stereomicroscope Pattern Evaluation (Fracture Pattern)

**Figure 3:**
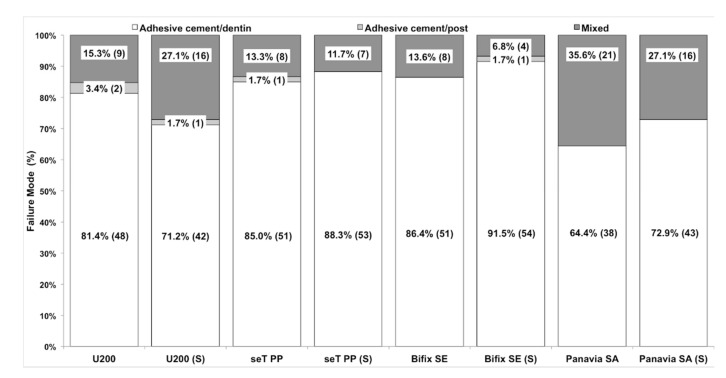
Percentage of fracture pattern (number of slices) of the groups evaluated.

In the fracture pattern analysis (Figure 3), all the cements presented predominantly cement/dentin adhesive failures, irrespective of the type of application.

### 3.3. Scanning Electron Microscopy (SEM)

**Figure 4:**
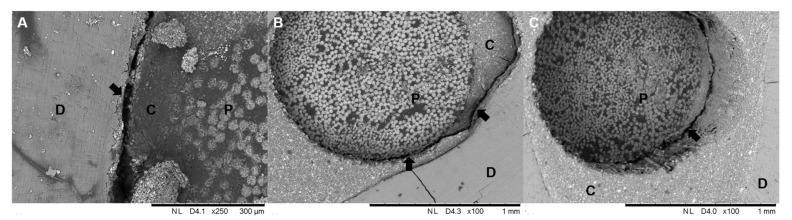
Representative SEM images of the failure modes observed in the study. (**A**) (250× magnification—Bar 300 µm): adhesive fracture between resin cement and root dentin (black arrow); (**B**) (100× magnification—Bar 1 mm): mixed fracture between post, resin cement and root dentin. Observe the fracture line extending along the interfaces (black arrows); (**C**) (100× magnification—Bar 1 mm): adhesive fracture between resin cement and post (black arrow). (D is Dentin, P is Post and C is Cement).

**Figure 5:**
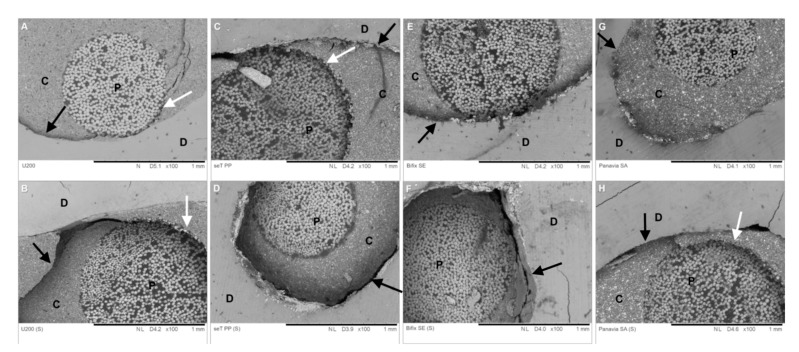
SEM images of the fractured specimens of push-out bond strength test. (**A**) (100× magnification—Bar 1 mm): U200 material—Centrix mode: a mixed fracture mode (adhesive cement/dentin and cement/post). It is possible to observe a fracture at the adhesive interface between the resin cement and the root dentin (black arrow), in addition to a fracture at the cement/post interface (white arrow). (**B**) (100× magnification—Bar 1 mm): U200 material—Sonic vibration: a mixed fracture (adhesive cement/dentin and cement/post). It is possible to observe a large quantity of resin cement on the dentin wall, in addition to a cohesive fracture in the resin cement (black arrow); (**C**) (100× magnification—Bar 1 mm): seT PP—Centrix mode: a mixed fracture mode (adhesive cement/dentin and cement/post). Fracture at the adhesive interface between the resin cement and the root dentin (black arrow), in addition to a fracture at the cement/post interface (white arrow). (**D**) (100× magnification—Bar 1 mm): seT PP—Sonic vibration: adhesive fracture cement/dentin (black arrow). (**E**) (100× magnification—Bar 1 mm): Bifix SE—Centrix mode: adhesive cement/dentin fracture mode (black arrow). (**F**) (100× magnification—Bar 1 mm): Bifix SE—sonic vibration: adhesive fracture (cement/dentin); (**G**) (100× magnification—Bar 1 mm): Panavia—Centrix mode: It is possible to observe a fracture between the resin cement and the root dentin (black arrow). (**H**) (100× magnification—Bar 1 mm): Panavia—Sonic vibration: a mixed fracture (adhesive cement/dentin and cement/post). It is possible to observe anadhesive fracture of the cement/post (white arrow)and cement/dentin interface (black arrow). (D is Dentin, P is Post and C is Cement).

Cohesive failures in dentin or cohesive failures in posts were not observed. Only fractures between adhesive cement/dentin (Figure 4a), post/cement (Figure 4b) and mixed were found (Figure 4c). SEM images, representative of the different types of fracture observed in the groups evaluated, are presented in Figure 5.

## 4. Discussion

The present study proposed the use of a sonic application technique as an alternative, with the purpose of improving the self-adhesive cement interaction with the dental substrate. However, in this study, the efficacy of the ultrasonic device was observed to be material dependent. Sonic application was more effective than the control application for the RelyX U200 and Bifix SE cements, while no difference was found for the Panavia SA and seT PP materials. Therefore, the null hypothesis proposed in this study was partially rejected.

With the purpose of reducing the operating stages and minimizing errors in bonding, self-adhesive resin cements with a chemical affinity for the dental structure have been used [5,18]. However, poor signs of demineralization on dentin could be observed when self-adhesive resin cement was used. Some studies have demonstrated the limited capacity of self-adhesive cements to demineralize and dissolve the smear layer in order to interact with the adjacent dentin [19,20]. This limitation is attributed to the high viscosity of the cement, which makes it difficult for it to penetrate into the dentin tubules [2,4,21]. The low penetration potential of acidic monomers in self-adhesive cements affects their ability to penetrate in the smear layer. This may interfere in hybrid layer formation, thereby compromising the bond of self-adhesive cements [20,22].

Some studies have demonstrated that the sonic application of adhesive materials may be an excellent alternative for improving the interaction of these materials with the mineral structure of dentin [11,13,14,23,24]. The present study demonstrated that the use of the ultrasonic technique improved the performance of the self-adhesive resin cements RelyX U200 and Bifix SE. Sonic vibration promoted a greater interaction and wettability of the dentin walls, by creating waves of pressure that facilitated the penetration of the material into the dentin substrate [13]. The Centrix syringe exerts no pressure on the cement, which may make its chemical and mechanical interaction with dentin difficult.

Furthermore, ultrasonic vibration may have had an influence on the thixotropic properties of the cementation agents and contributed to an increase in flow of the cement, improving the interaction of the cement with the substrate. An oscillating wave across the cement increases the fluidity, leading to a reduction in viscosity and consequently providing a greater power of diffusion [25]. Recently, one study showed that sonic devices resulted in films with fewer defects and that the use of sonic vibrations during the post accommodation process enhanced the cement flow [23]. Most authors investigating the effect of sonically applied dental material used ultrasonic devices. The positive effects of ultrasonic irrigation are based on cavitation, microstreaming and an increase of temperature. Ultrasonic devices range in frequency from 1000 (low-frequency ultrasonic) to 40,000 Hz [15]. The sonic device used in the present study had a mean frequency of 170 Hz and a direct comparison of the two methods is not possible. The good results of push-out bond strength under sonic application may be due to the increase in the degree of conversion of the material [13]. For this study, the authors justify the increase of the degree of conversion with the use of the sonic device is due to solvent evaporation of the adhesive system. On the other hand, the sonic movement can modify the kinetic energy of the material that would favor the increase of the degree of conversion. In the same way as it does with the use of sonic vibration in bulk-fill resins [26].

In its composition, RelyX U200 cement contains phosphoric acid ester and modified multifunctional methacrylate monomers, which have a high affinity for the minerals in the substrate. This may explain the better results of RelyXUnicem when compared with the resin cement Bifix SE [27]. 

Additionally, when Bifix SE was used for the cementation of zirconium crowns, it was capable of presenting a higher level of retention after one year, with good mechanical properties even after being submitted to stress for a long period [28]. Bifix SE is a cement that is more dependent on chemical activation than on light activation [29]. The sonic application may have been capable of accelerating and improving this chemical activation, and may have been responsible for the results found in this study.

Panavia SA has 10-MDP in its composition, which may have led to its good performance, irrespective of the mode of cement application. This monomer has a high chemical affinity for calcium; it is stable against hydrolysis and responsible for the interaction of the cement with hydroxyapatite [30]. The water solubility of 10-MDP-Ca salt was the lowest among the salts produced by the reaction between phosphoric acid ester monomers and apatite [31,32]. This monomer forms nanolayers at the bond interface, where the calcium ions released after partial dissolution of the hydroxyapatite bind together to form the 10-MDP-Ca bond, which is highly stable [33]. This chemical affinity is responsible for the low rates of dissolution of calcium salts, resulting in an optimum performance of these materials in the bond strength tests [31]. 

On the other hand, the lowest bond strength values were found for the cement seT PP, irrespective of the mode of application. For enamel and dentin conditioning to occur, and the later binding of the acidic groups to calcium and hydroxyapatite, the pH of a self-adhesive cement must initially be low when in contact with water and the humidity of the tooth. This will allow the formation of a stable bond between the methacrylate network and the dental structure [4]. However, the pH of seT PP remains very low after the reaction begins, which leads to the hydrophilicity of the cement and compromises its mechanical stability and its bond with the dentin substrate, thus reflecting negatively on its bond strength [34]. A recent study reported comparable microtensile bond strength values for seT PP and found this material demonstrated the lowest bond strength with multiple premature failures [20]. Some previous studies have reported that all specimens of seT PP cements failed before the test [34,35].

When analyzing the fracture pattern distribution of the self-adhesive cements, it was possible to observe a predominance of fractures of the cement/dentin adhesive type (Figure 2). When compared with the other cements, Panavia SA presented a higher number of mixed failures, irrespective of its mode of insertion into the root canal, which may indicate a low cohesive strength of the material. 

This study is the first regarding the use of a sonic application for self-adhesive resin cements in the cementation of GFPs, and, up to now, only studies with a sonic application of adhesive systems with good results have been evaluated.

## 5. Conclusions

The insertion of the self-adhesive cement into the root canal for the cementation of glass fiber posts using a sonic device promoted an increase in the bond strength values for the RelyX U200 and Bifix SE cements. The proposed sonic treatment may be considered as an alternative to amanual application in order to improve the bond strength of these cements.

## Figures and Tables

**Table 1:** Self-adhesive resin cement materials, manufacturer, lot number, composition, and application mode of the adhesive systems used.

Cements	Composition	Application Mode
**RELYX U200** **(3M/ESPE**, St.Paul, MN, USA) **Lot Number** **(1509000193)**	Base Paste: Silane-treated glass powder, 2-Propenoic acid, 2-methyl,1,1′-[1-(hydroxymethyl)-1,2-ethanodiyl] ester, triethylene glycol dimethacrylate (TEGDMA), silane-treated silica, glass fiber, sodium persulfate, and Tert-butyl peroxy-3,5,5-trimethylhexanoate. Catalyzer paste: Silane-treated glass powder, dimethacrylate substitute, silane-treated silica, Sodium p-toluenesulfonate, 1-Benzyl-5-phenylbarbituric acid, calcium salts, 1,12-Dodecanediol dimethacrylate, calcium hudroxide, and titanium dioxide.	1. Equal proportions of base and catalyzer pastes were dispensed on a glass plate and mixed with a metal spatula for 20 s; 2. Mixture was inserted into the root canal; 3. Light polymerization for 20 s.
**BIFIX SE** **(Voco GmbH, Cuxhaven, Germany)** **Lot Number** **(1504155)**	Base Paste: UDMA 25%, Bis-GMA 15%, Gli-DMA 10%, Kat N, N Bis 2%, Camphorquinone 1%, DABE 2%, BHT 0.1%, Glass Particles 39.9%, Silica 4.9%, and Ferric Oxide 0.1%. Catalyzer paste: UDMA 12%, Monomer BG 20%, Gli-DMA 20%, BHT 0.1%, DiHEMAPO4 1.9%, Glass Particles 38%, Silica 5%, BPO 50 FT 2%, and Titanium Dioxide 1%.	1. Equal proportions of base and catalyzer pastes were dispensed on a glass plate and mixed with a metal spatula for 20 s; 2. Mixture was inserted into the root canal; 3. Light polymerization for 20 s.
**seT PP** **SDI**, Bayswater, Australia) **Lot Number** **(S1407381)**	Phosphoric Methacrylate ester, UDMA, photoinitiator, Fluoroaluminosilicate, glass, and pyrogenic silica.	1. Equal proportions of base and catalyzer pastes were dispensed and the two pastes were mixed for 20 s; 2. Mixture was inserted into the root canal; 3. Light polymerization for 20 s.
**PANAVIA SA** (Kuraray Medical, Inc, Tokyo, Japan) **Lot Number** **(460039)**	Base Paste: 10-Methacryloyloxydecyl dihydrogen phosphate (MDP), bisphenol A diglycidyl methacrylate (Bis-GMA), Triethylene Glycol Dimethacrylate (TEGDMA), hydrophobic dimethacrylate, 2-hydroxymethylmethacrylate, (HEMA), silanated barium, Silanated colloidal silica, dl-Camphorquinone, peroxide, catalyzers, and pigments. Catalyzer paste: Hydrophobic aromatic dimethacrylate, Hydrophobic aliphatic dimethacrylate, silinated barium, sodium fluoride, accelerators, and pigments.	1. Equal proportions of base and catalyzer pastes were dispensed and the two pastes were mixed for 20 s; 2. Mixture was inserted into the root canal; 3. Light polymerization for 20 s.

Bis-GMA: bisphenol A diglycidyl ether methacrylate; HEMA: 2-hydroxyethylmethacrylate; TEGDMA: triethylene glycol dimethacrylate; BHT: 2,6-Di-tert-butyl-p-cresol; MDP: 10-Methacryloyloxydecyl dihydrogen phosphate; UDMA: urethane dimethacrylate.
